# Safety and Effectiveness of Motherwort Injection for Hemostasis and Menstrual Regulation: Protocol for a Prospective, Observational, Multicenter Real-World Study

**DOI:** 10.2196/103997

**Published:** 2026-11-11

**Authors:** Sicun Wang, Muzhi Li, Shuang Guan, Jun Liu, Haixia Dang, Zhong Wang, Yanan Yu

**Affiliations:** 1 Institute of Basic Research in Clinical Medicine, China Academy of Chinese Medical Sciences, 16 Nanxiaojie St, Dongzhimennei, Dongcheng District, Beijing, 100700, China, 86 15811215213; 2 China Academy of Chinese Medical Sciences, Beijing, China

**Keywords:** motherwort injection, protocol, real-world study, cohort study, obstetrics, gynecology

## Abstract

**Background:**

Motherwort injection (MI) is a traditional Chinese medicine injection widely used for hemostasis and menstrual regulation in obstetrics and gynecology. Although clinical trials and systematic reviews have demonstrated its effectiveness for specific indications, comprehensive real-world evidence (RWE) regarding its safety, effectiveness, and use patterns across diverse clinical settings remains limited.

**Objective:**

This study aims to evaluate the safety and effectiveness of MI in routine clinical practice and to generate RWE to support its rational use in women receiving treatment for approved obstetric and gynecological indications.

**Methods:**

This prospective, observational, multicenter real-world registry study plans to enroll 20,000 patients receiving MI for approved obstetric and gynecological indications in routine clinical practice in China. Treatment decisions, including whether to use MI and the dose and duration of treatment, as well as the use of concomitant medications, are made independently by treating physicians. Safety outcomes include the occurrence, type, severity, and incidence of adverse events and adverse drug reactions (ADRs). Effectiveness outcomes are assessed according to the clinical indication and include perioperative bleeding, postpartum hemorrhage, postabortion bleeding and menstrual resumption, uterine involution, menstrual regulation, and treatment satisfaction. Descriptive statistics will be used to characterize patient characteristics, MI use, and safety and effectiveness outcomes. Prespecified subgroup analyses will examine heterogeneity across major clinical groups. An exploratory nested case-control analysis with 1:4 matching and conditional logistic regression will be used to investigate factors associated with the occurrence of ADRs.

**Results:**

This study has received funding and obtained ethics approval in March 2024 (approval P24003-PJ03). Participant recruitment began on March 31, 2026. By September 2026, a total of 774 participants had been enrolled and included in the study database. The protocol specifies a target sample size of 20,000 participants, with data collection, monitoring, and quality control being conducted according to standardized study procedures. Data analysis has not yet commenced, and the study findings are expected to be published in October 2027.

**Conclusions:**

This study will provide large-scale RWE regarding the safety and effectiveness of MI in obstetric and gynecological practice. The findings are expected to support postmarketing safety surveillance, optimize clinical decision-making, and contribute to the evidence-based evaluation of traditional Chinese medicine injections.

## Introduction

Motherwort injection (MI) is a modernized herbal formulation derived from *Leonurus japonicus* (National Medical Products Administration [NMPA]; approval number Z51021448) and is administered intramuscularly. It has been widely used in China to manage various obstetric and gynecological conditions, including postpartum hemorrhage, postabortion hemorrhage, and menstrual disorders [1].

Previous studies have demonstrated that MI is effective in preventing and treating postpartum hemorrhage [2,3], promoting uterine involution [4], and facilitating the expulsion of retained placental membranes after childbirth [5]. In addition, MI has shown efficacy in reducing uterine bleeding after induced abortion and midtrimester pregnancy termination, while improving uterine recovery and coagulation function [6,7]. Furthermore, MI has been used to manage ovulatory dysfunction–related abnormal uterine bleeding and has been reported to reduce intraoperative and postoperative blood loss, as well as the duration of bleeding, during hysteroscopic surgery, myomectomy, and pregnancy termination procedures in patients with uterine fibroids [8-11].

Notably, these indications differ substantially in clinical settings, disease severity, treatment goals, and follow-up duration. For example, postpartum hemorrhage typically requires acute emergency intervention, whereas abnormal uterine bleeding and menstrual regulation often involve longer-term management. Despite these differences, most previous studies have focused on individual indications, and evidence regarding the safety and effectiveness of MI across multiple clinical scenarios remains limited. This heterogeneity may affect the generalizability of existing evidence and warrants further investigation.

Regarding safety, experimental studies have reported potential nephrotoxic effects [12-16], including lumbar pain, hematuria, renal tubular damage, and interstitial renal injury. Clinical studies have primarily identified mild-to-moderate adverse reactions, such as facial flushing, nausea, vomiting, tachycardia, chest distress, shortness of breath, fever, diarrhea, and injection-site induration [17]. In a postmarketing surveillance study involving 10,094 female patients, the reported incidence of adverse drug reactions (ADRs) was 0.79‰, and all reported ADRs were mild in severity [18].

Modern pharmacological studies have shown that motherwort contains active compounds such as leonurine and stachydrine, which regulate uterine smooth muscle contraction, promote endometrial repair, and exert anti-inflammatory effects [19]. Leonurine may facilitate endometrial repair by modulating gene and protein expression [20,21], whereas stachydrine may reduce uterine bleeding through immune regulation [22]. Motherwort also exhibits bidirectional effects on uterine smooth muscle contraction [23,24], promotes postpartum endometrial repair [25], inhibits inflammatory pathways related to menstrual regulation [26,27], and improves hemorheological function by reducing platelet aggregation and blood viscosity [28]. Existing studies generally suggest that motherwort has relatively low toxicity [29-31].

Although previous studies have provided evidence regarding the efficacy and safety of MI, most have focused on specific indications or selected patient populations, leaving uncertainty regarding its safety, effectiveness, and patterns of use across heterogeneous routine clinical settings. Real-world data (RWD) refer to data relating to patient health status and/or the delivery of health care routinely collected from various sources, whereas real-world evidence (RWE) refers to clinical evidence regarding the use and potential benefits or risks of a medical product derived from the analysis of RWD [32,33]. An RWE approach is particularly relevant to the present study because MI is used across multiple approved obstetric and gynecological indications that differ in patient characteristics, comorbidities, treatment objectives, concomitant therapies, and follow-up requirements. Compared with evidence generated in more controlled clinical settings, prospectively collected RWD can capture a broader and more heterogeneous spectrum of patients and treatment patterns, thereby improving the applicability of the findings to routine clinical practice. The prospective design also enables the longitudinal assessment of safety events and indication-specific clinical outcomes over clinically relevant follow-up periods without interfering with physician-directed treatment decisions. Conceptually, this study links patient characteristics and clinical indications with MI exposure patterns, concomitant treatments, prospectively collected safety events, and indication-specific effectiveness outcomes. This framework will enable the characterization of MI use patterns and safety outcomes in routine clinical practice, the evaluation of its effectiveness across relevant indication groups, and the exploration of patient- and treatment-related factors associated with ADR occurrence, thereby complementing evidence from conventional clinical studies.

Accordingly, building on a previous intensive hospital monitoring cohort involving 10,094 patients, this prospective, observational, multicenter real-world study aims to evaluate the safety and effectiveness of MI in a broader population. The primary objective is to characterize the frequency, categories, and types of adverse reactions and to explore factors associated with ADR occurrence to inform risk management. The study will also assess MI use patterns and indication-specific effectiveness outcomes, including outcomes related to hemorrhage control, uterine involution, and menstrual regulation, to provide evidence supporting the rational clinical use of MI and its associated risk management.

## Methods

### Study Design

This is a prospective, observational, multicenter, real-world registry study conducted at selected centers throughout China. The study has a target sample size of 20,000 patients with approved obstetric and gynecological indications for MI. This target was selected to improve the precision of estimates of rare ADRs, facilitate the identification of clinically relevant safety signals and factors associated with ADR occurrence, and support the evaluation of effectiveness across multiple approved indications. The detailed statistical rationale for the target sample size is provided in the Sample Size Estimation section.

The feasibility of large-scale recruitment is supported by the multicenter expansion strategy. Participant enrollment began on March 31, 2026, initially at 5 participating centers, where 530 participants were enrolled during the early recruitment phase. The study network has subsequently expanded to 15 participating centers, thereby substantially increasing the potential recruitment capacity. Recruitment progress will be regularly monitored across participating centers. The originally planned 1-year recruitment period represents an anticipated timeframe rather than a fixed termination point; recruitment will continue beyond 1 year, if necessary, until the prespecified target sample size of 20,000 participants is reached. Table 1 presents the schedule of study-related activities and assessments.

**Table 1.** Schedule of study procedures.

Procedures	Screening and enrollment	Treatment period	24-hour follow-up	7-day follow-up	1-year follow-up	Additional visits (if needed)
Eligibility confirmation	✓					
Baseline data (age, BMI, primary diagnosis, and indication)	✓					
Medical and medication history	✓					
Laboratory tests (if applicable)	✓					
Drug administration record (dose, route, and frequency)		✓				
Concomitant medication use		✓	✓	✓	✓	
Adverse events^a^		✓		✓		✓
Bleeding control after hysteroscopic surgery or uterine fibroid resection		✓			✓	
Postabortion recovery		✓			✓	
Postpartum hemorrhage		✓	✓			
Uterine involution		✓			✓	
Menstrual regulation		✓			✓	
Evaluation of treatment satisfaction		✓				
Recurrence or rebleeding events					✓	
Biological sample collection (if necessary)^a^						✓

a If an adverse event or adverse drug reaction (ADR) occurs, appropriate clinical measures will be taken. If the event remains unresolved at the end of the routine safety observation period, follow-up will continue as necessary to document its subsequent clinical course and final outcome. In the event of a serious adverse event (SAE) or suspected unexpected serious adverse reaction (SUSAR), motherwort injection (MI) treatment will be discontinued immediately, and appropriate clinical measures will be taken. If an SAE, SUSAR, or unexpected ADR occurs during the study, biological samples (eg, blood specimens) may be collected for additional analyses. Safety monitoring will be performed throughout MI treatment and daily for 7 days after discontinuation. Effectiveness outcomes will be assessed according to indication-specific outcome windows; therefore, not all participants will undergo the same long-term follow-up schedule.

### Study Setting and Center Selection

The study is conducted at participating health care institutions across China. According to the study protocol, the multicenter network is led by a tertiary grade A hospital qualified to conduct clinical drug research. Monitoring sites are selected from secondary-level or higher health care institutions across at least 3 provinces, autonomous regions, or municipalities, with consideration given to the clinical use of MI at potential participating institutions. Participants may be recruited from outpatient clinics and/or inpatient wards, and each participating center may include ≥1 clinical department as monitoring sites.

### Participants

Eligible participants are patients attending designated outpatient clinics and/or admitted to inpatient wards at participating centers who are judged by their physicians to have a clinical indication for MI and who voluntarily participate in the study in accordance with the ethics-approved study procedures. Patients who receive MI on more than 1 occasion, with an interval of ≥7 days between treatment episodes, will be considered new observation cases. The observation period for each participant will range from 7 days to 1 year, depending on the clinical indication and the participant’s individual condition.

### Intervention

This is an observational study that does not assign or modify any clinical intervention. The physician independently determines whether MI is clinically appropriate according to the patient’s clinical condition and routine clinical practice. For patients who are considered appropriate for and willing to receive MI, the physician determines the treatment regimen, including the dosage, treatment duration, and concomitant medications. Study participation does not determine or modify the clinical decision to use MI or the prescribed treatment regimen.

### Treatment and Follow-Up Procedures

The dosage and duration of MI treatment are determined by the treating physician. Safety monitoring begins with MI treatment. At the initial assessment, patient characteristics and medication information are recorded, followed by daily monitoring of medication use and safety information until 7 days after MI discontinuation. If no adverse event (AE) occurs, safety data collection is completed 7 days after the last MI administration. If a suspected AE occurs, the event is documented and followed according to the prespecified safety procedures. Effectiveness outcomes are assessed separately according to the assessment periods specified for each clinical indication. Thus, daily monitoring applies to the safety observation period rather than to the entire effectiveness follow-up period. The study procedure is shown in Figure 1.

**Figure 1.**
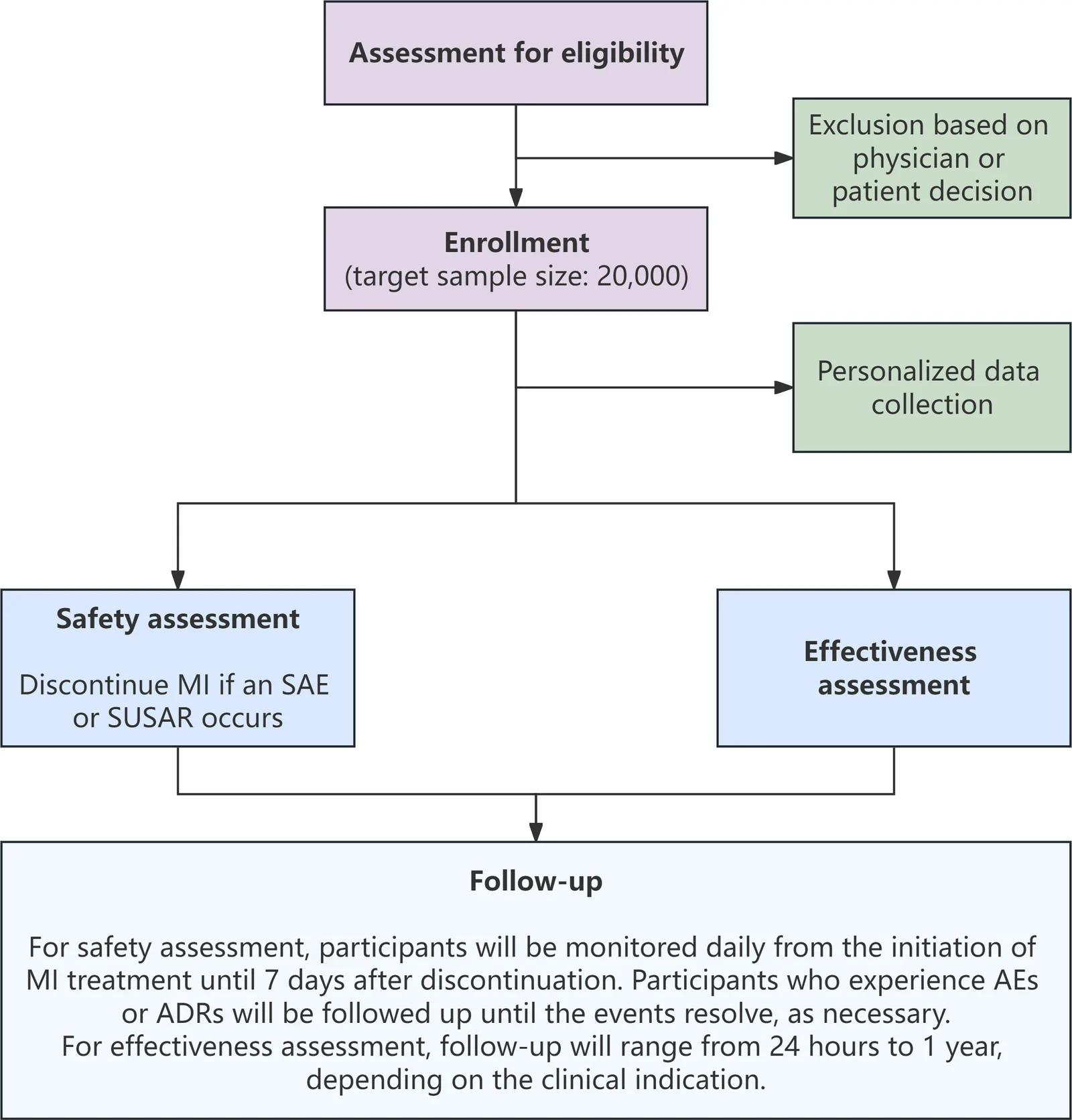
Study procedure. ADR: adverse drug reaction; AE: adverse event; MI: motherwort injection; SAE: serious adverse event; SUSAR: suspected unexpected serious adverse reaction.

Missing data may occur because participants decline to continue participation, experience AEs, or are lost to follow-up. To minimize missing data and their potential impact on safety evaluation, efforts will be made throughout the study to maintain participant follow-up and data completeness. If a participant is unable or unwilling to continue the scheduled follow-up, the research team will make reasonable efforts to contact the participant and, where feasible, complete a final follow-up visit. Available information related to the participant’s clinical course and study data will be followed up and documented as completely as possible to support the safety evaluation of MI.

### Sample Size Estimation

The primary objective of this study is not solely to detect the occurrence of at least 1 ADR but also to provide more precise estimates of ADR incidence and identify potential risk factors in real-world clinical practice.

A previous postmarketing surveillance study involving 10,094 patients reported an ADR incidence of 0.079%, which falls within the “rare” category according to the Council for International Organizations of Medical Sciences (CIOMS) classification criteria [34].

On the basis of the statistical principle known as the rule of 3 [35], approximately 3800 participants would be required to achieve a 95% probability of observing at least 1 ADR if the true incidence rate is 0.079%.

However, the present study is designed not only to improve the precision of ADR incidence estimates but also to detect clinically meaningful rare safety signals, evaluate effectiveness outcomes across multiple indications, and explore factors associated with ADR occurrence. Therefore, a larger sample size of 20,000 prospectively enrolled patients was considered appropriate.

In addition, the feasibility of the target sample size is supported by the multicenter recruitment strategy. Enrollment was initiated at 5 centers, and the study network has subsequently expanded to 15 participating centers. Recruitment will continue until the prespecified target sample size is achieved, even if the enrollment period extends beyond the originally anticipated 1-year timeframe.

### Outcome Measures

The primary outcomes include AEs, bleeding control following hysteroscopic surgery or uterine fibroid resection, postpartum hemorrhage, and postabortion recovery (Table 2). An AE is defined as any unfavorable medical occurrence after MI administration, including symptoms, signs, diseases, or laboratory abnormalities, regardless of whether a causal relationship with MI is established. AE severity will be graded according to the Common Terminology Criteria for Adverse Events (CTCAE), version 5.0 [36], from grade 1 (mild) to grade 5 (death related to the AE). AEs will be monitored daily from the initiation of MI treatment through 7 days after discontinuation.

**Table 2.** Overview of primary and secondary outcome measures and planned analyses.

Outcome categories and measures	Observation time	Planned analysis
Primary outcomes		
AEs^a^	Daily from the initiation of MI^b^ treatment through 7 days after discontinuation	Type, severity, and incidence of AEs
Bleeding control following hysteroscopic surgery or uterine fibroid resection	From the commencement of surgery until cessation of bleeding, with assessment extending up to 1 year	Intraoperative blood loss, postoperative blood loss, and cumulative bleeding duration
Postpartum hemorrhage	Within 24 hours after delivery	Incidence of postpartum hemorrhage and cumulative blood loss at 2 and 24 hours postpartum
Postabortion recovery	From completion of the abortion until cessation of bleeding or menstrual resumption, as applicable; assessed up to 1 year	Cumulative bleeding duration and time to menstrual resumption
Secondary outcomes		
ADRs^c^	Daily from the initiation of MI treatment through 7 days after discontinuation	Type and incidence of ADRs and exploratory analysis of associated factors
Menstrual regulation	From the initiation of medication until the return of normal menstruation or treatment completion, whichever occurs first; assessment extending up to 1 year	Treatment satisfaction
Uterine involution	From delivery until cessation of lochia, with assessment extending up to 1 year	Incidence of poor uterine involution and time to cessation of lochia
Treatment satisfaction	At completion of MI treatment	Physician- and participant-rated satisfaction using a 4-point score

a AE: adverse event.

b MI: motherwort injection.

c ADR: adverse drug reaction.

For each identified AE, information on its onset, clinical manifestations, severity, management, outcome, and causality assessment will be documented. If an AE remains unresolved at the end of the routine safety observation period, follow-up will continue as necessary to document its subsequent clinical course and final outcome. For bleeding control following hysteroscopic surgery or uterine fibroid resection, intraoperative blood loss, postoperative blood loss, and cumulative bleeding duration will be recorded. Postpartum hemorrhage is defined [37] as blood loss of ≥500 mL following vaginal delivery or ≥1000 mL following cesarean delivery within 24 hours after delivery, or blood loss accompanied by signs or symptoms of hypovolemia. The incidence of postpartum hemorrhage and cumulative blood loss at 2 and 24 hours postpartum will be assessed. For postabortion recovery, cumulative bleeding duration will be recorded as the number of days of vaginal bleeding after the procedure, and time to menstrual resumption will be defined as the number of days from completion of the abortion to the onset of the subsequent menstrual period.

The secondary outcomes include ADRs, menstrual regulation, uterine involution, and treatment satisfaction.(Table 2) An ADR is defined as a harmful or unintended response that is assessed as having at least a possible causal relationship with MI. Causality will be assessed according to the 2018 NMPA guidelines [38] and classified as definite, very likely, possible, possibly unrelated, pending evaluation, or unable to evaluate. Events classified as possible, very likely, or definite will be considered ADRs for the purposes of this study. The types and incidence of ADRs will be recorded, and factors associated with ADR occurrence will be explored. For menstrual regulation, particularly ovulatory dysfunction–related abnormal uterine bleeding, treatment effectiveness will be evaluated using treatment satisfaction. For uterine involution, outcomes will include the incidence of poor uterine involution and time to cessation of lochia; poor uterine involution is defined as persistence of lochia beyond 42 days postpartum. Overall treatment satisfaction will be assessed separately by physicians and participants at the end of MI treatment using a 4-point scale (0=“dissatisfied,” 1=“fair,” 2=“relatively satisfied,” and 3=“very satisfied”).

### Data Collection

Comprehensive participant characteristics and treatment information will be collected. Demographic and clinical data will include variables such as age, BMI, menstrual and obstetric history, medical history, allergy history, and ADR history. Clinical conditions including postpartum care, post–family planning procedures, menstrual disorders, and hysteroscopic interventions, as well as any comorbid conditions, will be assessed with a focus on the indications for MI. Detailed documentation of MI use will include whether it is the patient’s first use, the start and end dates of the current administration, the route of administration, dosage, injection site, batch number, and, if applicable, the reason for discontinuation. Furthermore, concomitant medications will be recorded, including the names of the drugs, their indications, and the start and end dates of their administration.

Data will be obtained from both routine clinical records and protocol-specified prospective data collection. Information available from medical and treatment records will be used whenever possible; any required information not available in these records may be supplemented through clinical observation, face-to-face interviews, telephone follow-up, or patient reporting. Authorized research personnel, including physicians and nurses, will record the required information using standardized paper-based or electronic study forms.

### Potential Bias and Mitigation Strategies

As a prospective observational study, this research may be subject to several potential sources of bias, including selection bias, information bias, confounding by indication, and bias due to loss to follow-up.

To minimize selection bias, consecutive eligible patients from participating centers will be enrolled. To reduce information bias, standardized investigator training, uniform case report forms, and centralized monitoring procedures will be implemented across all study sites.

Given the heterogeneity of clinical indications, important baseline variables—including age, indication type, disease severity, comorbidities, and concomitant medications—will be systematically collected to control for potential confounding. Prespecified subgroup analyses will be conducted to evaluate differences in safety and effectiveness outcomes across major patient groups.

Follow-up reminders, regular data verification procedures, and centralized monitoring will be used to minimize missing data and attrition bias.

Although treating physicians at participating centers are aware of the study, this awareness could potentially influence prescribing behavior in an observational setting. To minimize this possibility, the clinical decision to prescribe MI is made before consideration of study enrollment and remains entirely independent of study participation.

### Statistical Analyses

Data processing and statistical analyses will be performed using SAS (version 9.4; SAS Institute Inc). As this is a prospective observational study, descriptive statistical methods will be used primarily. Continuous variables will be summarized using the number of cases (n), mean and SD, median with IQR, and minimum and maximum values. Categorical variables will be summarized using frequencies and percentages. Baseline will be defined as the last nonmissing observation recorded prior to MI administration.

Safety outcomes will be summarized descriptively. The types and incidence of AEs and ADRs, as well as the severity of AEs, will be summarized using frequencies and percentages, as appropriate. Effectiveness outcomes will be analyzed according to the corresponding clinical indications and outcome types. Continuous outcomes, including blood loss, cumulative bleeding duration, time to menstrual resumption, and time to cessation of lochia, will be summarized using descriptive statistics. Categorical outcomes, including the incidence of postpartum hemorrhage, poor uterine involution, and treatment satisfaction ratings, will be summarized using frequencies and percentages.

Indication-specific subgroup analyses will be conducted for the major clinical indications for MI, including postpartum hemorrhage, postabortion recovery, bleeding associated with hysteroscopic surgery or uterine fibroid resection, uterine involution, and menstrual regulation. Within each indication subgroup, the corresponding effectiveness outcomes will be summarized using the descriptive methods specified above. Safety outcomes will also be summarized by clinical indication where the number of events is sufficient to provide meaningful estimates. These analyses are intended to describe potential heterogeneity in safety and effectiveness outcomes across clinical indications rather than to make causal comparisons between indication groups.

In addition, an exploratory nested case-control analysis will be conducted within the registry cohort to further investigate factors associated with ADR occurrence. Cases will include female patients who experience ADRs during MI treatment and whose events are assessed as “possible” or higher according to the causality assessment criteria. For each ADR case, 4 patients who received MI but did not experience ADRs during the same observation period will be selected as matched controls.

Matching variables will include indication type, age (within ±3 years), study center, and relevant uterine conditions. For patients with postpartum or postabortion bleeding, additional matching factors may include parity, scarred uterus status, singleton or multiple pregnancy, and placenta previa status, when applicable.

A 1:4 matching strategy will be adopted. After matching, conditional logistic regression analysis will be performed to evaluate factors associated with ADR occurrence. Propensity score methods may be used as supplementary analyses to further assess residual confounding. Sensitivity analyses will be conducted to assess the potential influence of missing values, outliers, and other influential data. Detailed procedures for the sensitivity analyses will be specified in the statistical analysis plan before database lock.

### Data Quality Control

A kick-off meeting will be convened at the lead institution, followed by comprehensive training sessions for relevant personnel at participating subcenters. Participants in these sessions will include principal investigators, researchers, monitors, and monitoring assistants from each center. The primary objectives of the training will be to elucidate the study workflows, delineate the responsibilities of each team member, and standardize the completion of documentation forms.

All trainees are required to undergo competency assessments to verify their comprehension of the study-related knowledge, mastery of the requisite skills, and ability to execute their assigned roles effectively. The results of these assessments, along with associated records, constitute essential components of the study documentation and will be securely archived for future reference.

### Data Management

This study uses an electronic data capture (EDC) system to enhance the efficiency of data management processes. The system administrator creates distinct user accounts for various stakeholders, including patients, investigators, monitors, auditors, and data managers, assigning role-specific access permissions to ensure data confidentiality. Data entered into the EDC system are considered source data unless explicitly indicated otherwise; nonsource data must align with original records to ensure consistency and accuracy. It is imperative that all observations and test results are recorded promptly, accurately, completely, and authentically within the EDC system, with any unauthorized modifications strictly prohibited. Any data corrections require a justification for the modification, which must be documented through system prompts. All fields in the EDC system are mandatory, and omissions or blank entries are not allowed. Monitors will review all data collection forms for completeness and accuracy.

For data deemed questionable, authorized personnel (data managers, clinical research associates, and other qualified staff) are empowered to send queries to investigators or patients through the system. Investigators or patients are required to confirm, explain, or rectify queried data. The initiator of the query will evaluate the responses and either mark the query as resolved or escalate it, if necessary. An audit trail of all queries will be maintained to ensure adherence to regulatory requirements.

The database will be locked once all data have been entered, all queries have been resolved, analysis populations have been defined and validated, and the statistical analysis plan (SAP) has been finalized. The locked database will be securely archived and transmitted to statisticians in specified formats for subsequent analysis.

### Ethical Considerations

The study protocol was reviewed and approved by the ethics committee (EC) of the Institute of Basic Research in Clinical Medicine, China Academy of Chinese Medical Sciences (CACMS), in March 2024 (P24003-PJ03). The ethics approval certificate will be provided as supplementary material.

This study will be conducted in accordance with the Declaration of Helsinki, Good Clinical Practice (GCP) guidelines, and relevant regulatory requirements for postmarketing safety reevaluation of traditional Chinese medicine injections (TCMIs). As this is a real-world observational study that does not interfere with routine clinical care and poses no more than minimal additional risk to participants, the EC approved a waiver of the requirement for signed written informed consent. However, the waiver does not exempt the study from informing participants about the research. At enrollment, patients will be informed about the study, and only those who agree to the collection of study-related information will be enrolled. Thus, participants are informed about the study and their agreement to study-related data collection is obtained, but they are not required to sign a written informed consent form.

If biological samples are required for the investigation of suspected unexpected serious adverse reactions (SUSARs), additional written informed consent will be obtained from the participant prior to sample collection and centralized testing.

## Results

This study is supported by grants from the Independent Research Fund of the CACMS (grant ZB2025024) and the Fundamental Research Funds for the Central Public Welfare Research Institutes (grant ZZ13-YQ-076). The study protocol was approved by the EC of the CACMS in March 2024 (approval number P24003-PJ03). Participant enrollment began on March 31, 2026. By September 2026, a total of 774 participants had been enrolled and entered into the study database. The study initially involved 5 participating centers, and the number of centers increased to 15 by June 5, 2026. The study aims to prospectively enroll 20,000 participants. Although the original protocol anticipated an enrollment period of approximately 1 year, recruitment will continue beyond this period if necessary to achieve the prespecified target sample size. Data collection and follow-up will proceed according to the observation periods specified for the safety and indication-specific effectiveness outcomes. Database cleaning, data quality assessment, and statistical analyses will be conducted after completion of the corresponding data collection procedures. No formal analyses of safety or effectiveness outcomes are reported in this protocol. The study findings are expected to be published in October 2027.

## Discussion

### Study Design Features

TCMIs represent a significant advancement in the modernization of traditional Chinese medicine and are characterized by rapid onset of action and high bioavailability [39]. As complex compound formulations, TCMIs exert therapeutic effects through multiple targets and pathways, which contribute to their clinical effectiveness but also pose challenges for comprehensively evaluating their safety and effectiveness. Therefore, scientifically rigorous postmarketing surveillance and reevaluation are essential to support their rational use in clinical practice.

MI is widely used in obstetric and gynecological practice for approved indications, including the prevention of postpartum hemorrhage, the promotion of uterine recovery after delivery or abortion, and the management of menstrual disorders. Given the diversity of clinical indications and patient characteristics, this prospective, multicenter real-world study is designed to enroll a large sample of patients from routine clinical practice. Data collection and quality control procedures will be conducted throughout the study period to ensure the completeness and reliability of the study database.

### Strengths of the Study Design

To our knowledge, this study is the largest prospective real-world study of MI conducted to date and is the first to systematically evaluate its use across multiple approved indications within a unified research framework. Previous studies have primarily focused on individual indications, including postpartum hemorrhage and abortion-related conditions [2,3,40]. By enrolling a large and diverse patient population, this study is expected to generate comprehensive evidence regarding the safety profile, effectiveness, and use patterns of MI in routine clinical practice.

In addition to evaluating ADRs and clinical outcomes, this study incorporates physician- and patient-reported assessments of treatment satisfaction, providing a broader understanding of treatment experiences from the patient perspective. Furthermore, the systematic analysis of safety and effectiveness outcomes may contribute to the development of an objective benefit-risk assessment framework for MI. The findings are expected to strengthen pharmacovigilance efforts and provide evidence to support clinical decision-making and regulatory evaluation of MI.

### Limitations of the Research Methods

As a real-world observational study, this study has several anticipated limitations. First, despite the implementation of predefined quality control procedures and statistical adjustment methods, residual confounding and selection bias cannot be completely eliminated because treatment allocation is determined by routine clinical practice rather than randomization. Second, the inclusion of multiple approved indications may introduce clinical heterogeneity in patient characteristics, treatment objectives, and outcome measures, which should be considered when interpreting the study findings.

### Conclusions

In conclusion, the pragmatic, observational design of this study, which encompasses a patient population representative of real-world MI users, is expected to generate prospective data on the safety and effectiveness of MI across a broad cohort of patients with obstetric and gynecological conditions. This approach is anticipated to offer enhanced insight into the real-world application and outcomes of MI.

## Supplementary material

10.2196/103997 Checklist 1 STROBE checklist.

## Abbreviations

ADR: adverse drug reaction
AE: adverse event
CACMS: China Academy of Chinese Medical Sciences
CIOMS: Council for International Organizations of Medical Sciences
CTCAE: Common Terminology Criteria for Adverse Events
EC: ethics committee
EDC: electronic data capture
GCP: Good Clinical Practice
MI: motherwort injection
NMPA: National Medical Products Administration
RWD: real-world data
RWE: real-world evidence
STROBE: Strengthening the Reporting of Observational Studies in Epidemiology
SUSAR: suspected unexpected serious adverse reaction
TCMI: traditional Chinese medicine injection
