# Supplementary material for: Safety and Effectiveness of Motherwort Injection for Hemostasis and Menstrual Regulation: Protocol for a Prospective, Observational, Multicenter Real-World Study
Source: JMIR Res Protoc. 2026 Nov 11;15:e103997. doi: 10.2196/103997 (PMC13754181; doi:10.2196/103997)
Supplement: Supplementary file 1 [file resprot-v15-e103997-s001.pdf]

STROBE Statement—Checklist of items that should be included in reports of ***cohort studies***

	Item No	Recommendation	Reported on Page No
**Title and abstract**	1	(*a*) Indicate the study's design with a commonly used term in the title or the abstract	Title, p. 1; Abstract, pp. 3-4
		(*b*) Provide in the abstract an informative and balanced summary of what was done and what was found	Abstract, pp. 3-4
**Introduction**			
Background/rationale	2	Explain the scientific background and rationale for the investigation being reported	Introduction, pp. 5-8
Objectives	3	State specific objectives, including any prespecified hypotheses	Introduction, p. 8
**Methods**			
Study design	4	Present key elements of study design early in the paper	Study design, pp.8- 9
Setting	5	Describe the setting, locations, and relevant dates, including periods of recruitment, exposure, follow-up, and data collection	Study design, pp. 8-9; Study setting and center selection, p. 13
Participants	6	(*a*) Give the eligibility criteria, and the sources and methods of selection of participants. Describe methods of follow-up	Participant, p. 13; Treatment and follow-up procedures, p. 14
		(*b*) For matched studies, give matching criteria and number of exposed and unexposed	Statistical analyses, pp. 22-23
Variables	7	Clearly define all outcomes, exposures, predictors, potential confounders, and effect modifiers. Give diagnostic criteria, if applicable	Intervention, pp. 13-14; Outcome measures, pp. 16-18; Potential bias and mitigation strategies, p. 21
Data sources/ measurement	8*	For each variable of interest, give sources of data and details of methods of assessment (measurement). Describe comparability of assessment methods if there is more than one group	Outcome measures, pp. 16-18; Data collection, p. 20
Bias	9	Describe any efforts to address potential sources of bias	Potential bias and mitigation strategies, p. 21
Study size	10	Explain how the study size was arrived at	Study design, pp. 8-9; Sample size estimation, pp. 15-16
Quantitative variables	11	Explain how quantitative variables were handled in the analyses. If applicable, describe which groupings were chosen and why	Statistical analyses, pp. 22-23
Statistical methods	12	(*a*) Describe all statistical methods, including those used to control for confounding	Statistical analyses, pp. 22-23
		(*b*) Describe any methods used to examine subgroups and interactions	Statistical analyses, pp. 22-23
		(*c*) Explain how missing data were addressed	Treatment and follow-up procedures, p. 14; Potential bias and mitigation strategies, p.

			21;<br>Statistical analyses, pp. 22-23
		(*d*) If applicable, explain how loss to follow-up was addressed	Treatment and follow-up procedures, p. 14; Potential bias and mitigation strategies, p. 21;
		(*e*) Describe any sensitivity analyses	Statistical analyses, pp. 22-23
**Results**			
Participants	13*	(a) Report numbers of individuals at each stage of study—eg numbers potentially eligible, examined for eligibility, confirmed eligible, included in the study, completing follow-up, and analysed	Results, pp. 26-27 (recruitment progress only, full participant flow will be reported after study completion)
		(b) Give reasons for non-participation at each stage	Not applicable
		(c) Consider use of a flow diagram	Figure 1, p. 15 (study procedure). Full participant flow diagram to be reported after study completion
Descriptive data	14*	(a) Give characteristics of study participants (eg demographic, clinical, social) and information on exposures and potential confounders	Not applicable
		(b) Indicate number of participants with missing data for each variable of interest	Not applicable
		(c) Summarise follow-up time (eg, average and total amount)	Not applicable
Outcome data	15*	Report numbers of outcome events or summary measures over time	Not applicable
Main results	16	(*a*) Give unadjusted estimates and, if applicable, confounder-adjusted estimates and their precision (eg, 95% confidence interval). Make clear which confounders were adjusted for and why they were included	Not applicable
		(*b*) Report category boundaries when continuous variables were categorized	Not applicable
		(*c*) If relevant, consider translating estimates of relative risk into absolute risk for a meaningful time period	Not applicable
Other analyses	17	Report other analyses done—eg analyses of subgroups and interactions, and sensitivity analyses	Statistical analyses, pp. 22-23, results not yet available
**Discussion**			
Key results	18	Summarise key results with reference to study objectives	Not applicable
Limitations	19	Discuss limitations of the study, taking into account sources	Limitations of the

		of potential bias or imprecision. Discuss both direction and magnitude of any potential bias	research methods, p. 28
Interpretation	20	Give a cautious overall interpretation of results considering objectives, limitations, multiplicity of analyses, results from similar studies, and other relevant evidence	Not applicable
Generalisability	21	Discuss the generalisability (external validity) of the study results	Introduction, pp. 7–8;
**Other information**			
Funding	22	Give the source of funding and the role of the funders for the present study and, if applicable, for the original study on which the present article is based	Funding, p. 31

*Give information separately for exposed and unexposed groups.

Note: An Explanation and Elaboration article discusses each checklist item and gives methodological background and published examples of transparent reporting. The STROBE checklist is best used in conjunction with this article (freely available on the Web sites of PLoS Medicine at http://www.plosmedicine.org/, Annals of Internal Medicine at http://www.annals.org/, and Epidemiology at http://www.epidem.com/). Information on the STROBE Initiative is available at http://www.strobe-statement.org.
